# Static Balance Modification during the Workday in Assembly Chain Workers with and without Current Low Back Pain

**DOI:** 10.3390/ijerph17207385

**Published:** 2020-10-10

**Authors:** Ana Vanessa Bataller-Cervero, Cristina Cimarras-Otal, Luis Enrique Roche-Seruendo, Andrés Alcázar-Crevillén, José Antonio Villalba-Ruete, César Berzosa

**Affiliations:** 1 Facultad de Ciencias de la Salud, Universidad San Jorge, 50830 Villanueva de Gállego, Spain; avbataller@usj.es (A.V.B.-C.); leroche@usj.es (L.E.R.-S.); cberzosa@usj.es (C.B.); 2 Laboratorio de Biomecánica, Hospital MAZ, 50015 Zaragoza, Spain; aalcazar@maz.es; 3 Servicio Médico, BSH/EE, 50016 Zaragoza, Spain; jose-antonio.villalba@bshg.com

**Keywords:** lumbago, equilibrium, pressure platform, manufacturing workers

## Abstract

Background: Low back pain (LBP) is a common recurrent pathology among assembly chain workers. This population tends to spend most of the workday in a static standing posture and handling loads, with balance being essential for correct job performance. LBP is related to poorer postural control, so balance could be affected in this condition. Methods: The purpose of the present study is to analyze the deterioration of static balance generated by work activity in a prolonged standing position. We assess sway with a pressure platform at three moments of the workday (before, during, and after work), comparing the different balance parameters in 22 manufacturing plant workers with (17) and without (5) LBP. Results: In the pre-work capture, an independent *t*-test showed no significant differences between the pain and non-pain groups’ static balance parameters. Between the pre- and mid-workday captures, a two-way ANOVA with repeated measures showed a significant decrease in the medial-lateral center of pressure displacement with open eyes in workers with LBP. Conclusions: workers with low back pain do not show a greater deterioration in static balance than workers without pain during the workday.

## 1. Introduction

Musculoskeletal disorders (MSDs) affect millions of European workers every year in all types of job and employment sectors, with the most prevalent at the European level being occupational disease. According to Eurostat figures on recognized occupational diseases, MSDs are the most common occupational disease (39% of the total) [1]. Among the different forms of MSD, low back pain is a common recurrent problem among workers, with many sickness absence episodes registered [1], and the manufacturing industry is also affected by this kind of sickness leave [2]. Moreover, manufacturing workers habitually spend most of their working day in a standing posture, making repetitive movements with their upper limbs. Static positions, besides awkward lifting movements, can be mechanical causes of low back pain in the workplace [3,4]. Recent studies claim that low back pain in blue collar workers is correlated with long bouts of time (>10 min) spent in standing positions [5]. Furthermore, standing for prolonged periods has been shown to cause fatigue and low back discomfort, although the causes are not yet clear [6]. In addition, low back pain has a high prevalence in metal-working environments [7,8,9] and in other employment sectors, such as health work [10,11,12] or catering [13]. Their work is related to repetitive movements with awkward postures or prolonged static postures that could cause or exacerbate these pathologies.

People with low back pain (LBP) have poorer postural control compared to healthy people. Several studies have shown an association between the LBP registered by different questionnaires such as the Oswestry Low Back Pain Disability Questionnaire and an alteration in sway parameters [14,15,16]. Increased postural sway is also commonly found in patients suffering from this musculoskeletal disorder [17,18]. Additionally, an increased postural instability can be found in people suffering from LBP, with this instability deteriorating with the pain level (16). Several theories have been developed in an effort to explain this effect. One of them holds that postural stability may be affected by damage to the sensory tissues in the lumbar spine, reducing the accuracy of the sensory integration process that functions to estimate the position of the body and make the corresponding adjustments [19]. Other theories try to explain the increased postural sway by the decreased muscle control caused by the pain, with nociceptive afferents pathways that could interfere with the spinal motor ones [20]. 

Postural sway can be measured by registering the deviations in the location of the center of pressure (COP) by means of a force or pressure platform. The COP is the point that reflects the pressure of the body over the feet in one spot. The body should be able to generate quick COP transitions that just exceed the current position of the center of mass (COM) and accelerate it in the opposite direction in order to maintain balance [21]. As previously said, the proprioceptive system of LBP patients is altered due to the pain. Subjects with LBP present a delay in the action of the stabilizing muscles of the back [22]. The accentuation of the loss of balance with closed eyes would be explained by the greater demand that this would cause in the patient, since having their proprioceptive system altered and a slower reaction speed is more likely to cause worse posture control [23]. Therefore, the Romberg test, which compares the balance in open and closed-eyes conditions, might be interesting as a screening tool [24]. 

Most studies analyze the effect of prolonged standing on the balance in LBP and healthy workers outside their real workday tasks, simulating prolonged standing conditions. Therefore, the purpose of this study is to assess the postural balance in workers with and without LBP at the moment of the evaluation, studying the relationship of the balance with the working time at a specific job activity in a manufacturing assembly chain. It was hypothesized that workers with current LBP will show a worse static balance and a larger deterioration in standing balance during the workday compared to workers without low back pain.

## 2. Materials and Methods 

### 2.1. Subjects

Twenty-two workers between 26 and 60 years old employed at a Spanish manufacturing company voluntarily participated in the study. The age of the subjects was selected due to the fact that, on one hand, they are of working age, and on the other hand the possible effect of the age in the balance characteristics is not still present [25,26]. From the age of 60, the balance deteriorates, and this could affect the study conclusions. 

They all worked in different sections of the assembly line, spending most of the working day in a standing position. The duration of the working day is 8 hours for the morning shift, with two breaks of 20 and 10 minutes, respectively. 

It was decided to consider for the study workers who had suffered a previous episode of LBP in the last two years but without a current limitation in their work. In the sample of the study, we expected to find a group of workers with LBP, as this is a pathology where episodes of pain are recurrent and could be chronic. Thus, the inclusion criteria for participation in this study were to have suffered an absence from work based on LBP diagnosis in the past two years, but without any current restriction at work. Twenty-two workers met the inclusion criteria and were considered for the analysis. The exclusion criteria were to be limited in their work at the time of the study. All the participants signed informed consent. This study was approved by the University Ethical Committee and was conducted in compliance with the ethical principles for research involving human subjects expressed in the Declaration of Helsinki [27].

### 2.2. Experimental Design and Procedure

The subjects indicated their LBP level at the beginning of the workday using a six-point numerical rating scale (NRS-6) with values from 0 (no pain) to 5 (maximum pain) [28]. The workers were divided into two groups depending on the pain level indicated: a pain group (PG) of workers with a level of 1 or more on the NRS-6 and a non-pain group (NPG) of workers with a value of zero. The workers were also asked about their working shift, years of experience, and weight and height so that their body mass index (BMI) could be calculated. 

The static postural balance of the workers enrolled in this study was measured at three points in the workday: before the start of the workday, in the middle, and after finishing it. A quiet dedicated room in the medical service of the company was used for the study. The postural balance was measured with a Freemed baropodometric pressure platform (Sensor Medica, Rome, Italy) composed of a pressure-sensitive plate with an active surface of 400 × 400 mm and that was 8 mm thick. The FreeStep v.1.0.3 software (Sensor Medica, Rome, Italy) was used to record and analyze the data. The reliability of this baropodometric platform has been shown in other studies [29]. 

In the three trials, the participants were instructed to stand as still as possible for 51.2 s in a barefoot standing position with feet approximately at pelvis width, looking straight ahead and keeping their arms at their sides in a comfortable position. The effect of the foot position on the sway parameters has been analyzed in previous studies, concluding that these parameters are not different in the preferred foot positions from several proposed foot positions. The self-selected position could be considered more natural and takes into consideration the body anthropometry [30].

The proprietary software of the platform suggests this sampling time as the value used in previous investigations [29]. The recordings of postural balance were conducted in two conditions: with open eyes (OE) looking at a visual target adjusted at the height of the eyes at a distance of about 1.5 m, and with closed eyes (CE). The measurement protocol was as follows: rest on the platform (20 s), acquisition in the OE condition (51.2 s), rest (10 s), and acquisition in the CE condition (51.2 s). 

This test measures the center of pressure (COP) position in the antero-posterior (AP) and medial-lateral directions. The greater the values, the worse the postural stability. The protocol also measures the sway area, which is quantified as 90% of the total area covered by the COP trajectory in the ML and AP directions using an ellipse to fit the data. The smaller the surface, the better the postural balance. The length covered by the COP over the support base throughout the sampling time is the mean velocity. The mean velocity reflects the efficiency of the postural control system (the smaller the velocity, the better the postural control). Other parameters to analyze are the COP root mean square displacements in the antero-posterior (RMS-AP) and medial-lateral directions (RMS-ML). RMS is defined as the square root of the mean of the squares of a sample. This is an index of variability of COP movements and offers good reliability in discriminating healthy subjects from those with pathologies [31]. The Romberg coefficient was calculated as the COP sway length in OE by the sway length in CE conditions. A lower coefficient indicates a higher use of visual references in order to maintain balance. All these variables were calculated with the pressure platform proprietary software FreeStep. Among all the variables, the COP mean velocity (mean velocity) has been demonstrated as variable, but generally has a good reliability [32]. 

### 2.3. Statistical Analysis

The normality of the variables was assessed by the Shapiro–Wilk test. An independent *t*-test was performed to assess the group effect in the COP variables in the pre-workday evaluation between the PG and NPG workers. A two-way ANOVA with a repeated measures test was applied with a polynomial a priori test statistic in order to analyze the differences in COP pattern parameters measured at the three times of the workday (pre-workday, mid-workday, and post-workday) for the two groups (PG, NPG). If significant differences were found for the variable repetition, a one-way ANOVA with repeated measures for the PG and NPG was performed with a post-hoc Bonferroni adjustment.

In the case of non-parametric variables, the Friedman test was applied with a Dunn’s test for the individual comparison between the three times of the workday.

For statistical analysis purposes, SPSS version 21.0 (SPSS, Inc., Chicago, IL, USA) was applied, conducting the graphical representation with Graphpad Prism v.7.03 software (GraphPad Software San Diego, CA, USA). All the results are presented as means ± standard deviations, and the effect size (ES) was evaluated with the d of the Cohen statistic, where the threshold values were >0.2 (small), >0.6 (moderate), and >1.2 (large) [33]. A *p* value of 0.05 was set as the criterion for statistical significance. 

## 3. Results

### Subjects

Twenty-two workers were considered for the study, all from the same plant of an international company, with 3 women and 19 men in the sample. The workers were 43 ± 7 years of age, with a height of 175.2 ± 8.9 cm and a weight of 80.8 ± 12.2 kg (Table 1). 

Among all the workers who participated in the study, 17 workers referred to LBP in the previous workday evaluation (PG) and five workers did not indicate LBP (NPG). No significant differences were found in the parameters of the test in the pre-workday COP assessment. However, several COP parameters present a medium effect size: ML_OE (ES = 0.92), RMS-AP_OE (ES = 0.74), and RMS_ML_CE (ES = 0.62) (Table 2).

**Figure 1:**
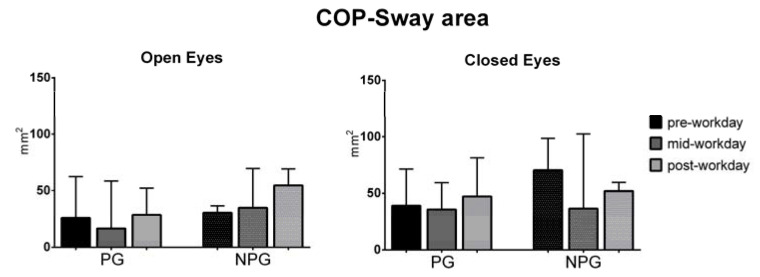
Median with interquartile range of the COP sway area in open-eyes (OE) and closed-eyes (CE) conditions in pre-workday, mid-workday, and post-workday assessments.

**Figure 2:**
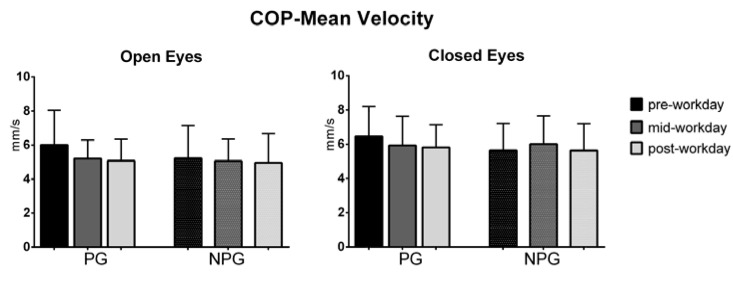
Mean with standard deviation of the COP mean velocity in open-eyes (OE) and closed-eyes (CE) conditions in pre-workday, mid-workday, and post-workday assessments.

**Figure 3:**
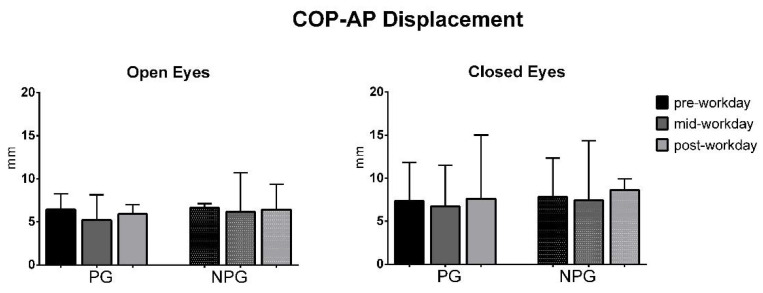
Median with interquartile range of the COP antero-posterior (AP) displacement in open-eyes (OE) and closed-eyes (CE) conditions in pre-workday, mid-workday, and post-workday assessments.

**Figure 4:**
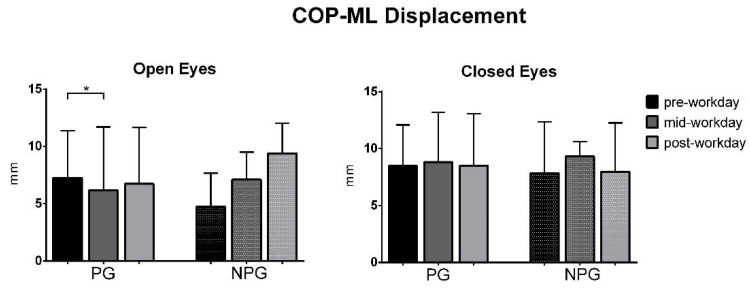
Median with interquartile range of the COP medial-lateral displacement in open-eyes (OE) and closed-eyes (CE) conditions in pre-workday, mid-workday, and post-workday assessments. The symbol * is the significant difference found in Pain Group between the first and second measurement.

**Figure 5:**
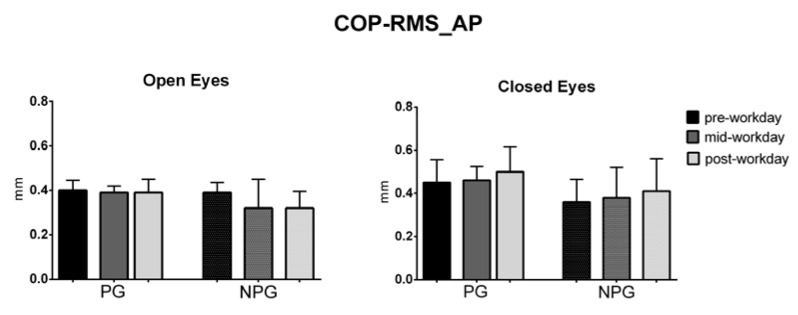
Median with interquartile range of the COP root mean square in antero-posterior direction in open-eyes (OE) and closed-eyes (CE) conditions in pre-workday, mid-workday, and post-workday assessments.

**Figure 6:**
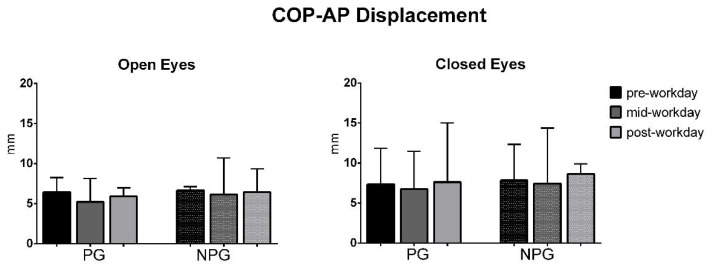
Median with interquartile range of the COP root mean square in medial-lateral direction in open-eyes (OE) and closed-eyes (CE) conditions in pre-workday, mid-workday, and post-workday assessments.

The ANOVA test of the COP parameters before the workday (pre-workday), in the middle of the workday (mid-workday), and after finishing it (post-workday) in the open and closed-eyes conditions showed statistically significant differences in the COP displacement only in the medial-lateral direction in the OE condition in PG workers (*p* = 0.01) (Figure 1, Figure 2, Figure 3, Figure 4, Figure 5 and Figure 6).

**Figure 7:**
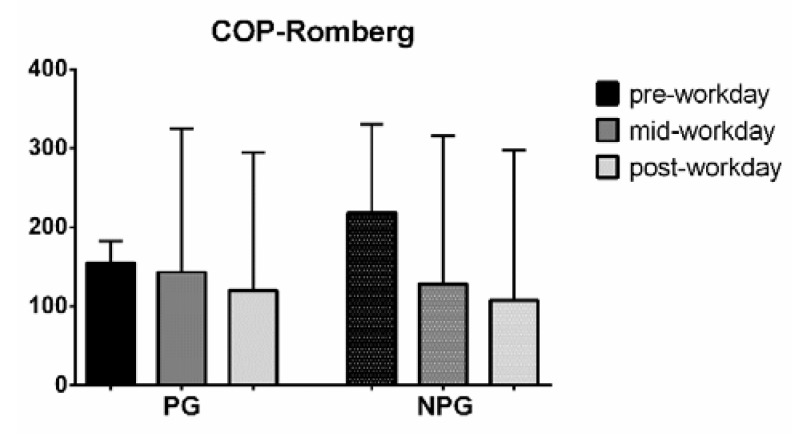
Median with interquartile range of the COP Romberg coefficient in pre-workday, mid-workday, and post-workday assessments.

A study of the visual contribution to balance by means of the Romberg coefficient for the NPG shows a decrease in the Romberg coefficient during the workday in both PG and NPG (Figure 7).

## 4. Discussion

The aim of the present study was to compare the COP parameters between workers with and without current LBP and their evolution during the workday.

### 4.1. Balance and Low Back Pain

According to the results of the measurements in the pre-workday capture, it cannot be affirmed that workers with LBP had worse balance than those without back pain at the time of the study. None of the balance parameters analyzed show significant differences between the groups, although it can be seen that with open eyes all the parameters are higher in subjects with low back pain. A larger sample might make this difference significant. 

The results of the literature do not offer clear conclusions on the subject, but the general trend is towards an increased AP and RMS-AP sway in LBP subjects. Mann et al. [34], analyzing the balance in healthy young women and women with LBP in OE and CE conditions, found a significantly larger value in the AP and ML displacement in the LBP group in both conditions. Additionally, in [35], the position of the COP in AP showed a more pronounced value in LBP patients, which could be explained by a lack of extension of the knees in order to relieve back pain. Harringe et al. [36] only found significant differences in the COP area in gymnasts with and without LBP when measuring balance over a foam surface. As the authors suggest, low back pain may cause the stiffening of the spine, which may lead to an ankle strategy and a possible increased AP sway in this group.

Additionally, a higher COP sway velocity is usually found in LBP cases. Da Silva et al. [17] found a higher mean velocity and sway area in workers with LBP vs. healthy workers. Similar results have been found in other studies [14,37]. 

In contrast, only a few studies have found a lesser value of sway for subjects with LBP [38,39]. The explanation offered for these results corresponds to a modern theory suggesting that psychological aspects of pain could lead to more rigid COP excursion, which could have indicated a more stable and healthier system of postural control [40]. 

### 4.2. Balance Evolution during Workday

Studying the evolution of the COP parameters during the workday, a statistically significant decrease in the COP-ML displacement is only found in the open-eyes condition between the pre-workday and mid-workday captures in PG. These results do not support our hypothesis of the deterioration of COP parameters due to workday fatigue, with a higher degree for PG than for NPG workers. 

In line with our hypothesis not being proved, Lafond et al. [41] found that LBP subjects showed an increased COP RMS and COP mean velocity after a 30 min standing task. A study of COP variations during 2 hours in a standing position shows an increase in AP displacement in men with LBP after 15 min, with a gradual increase over time [42]. 

In our study, a non-significant decrease in mean velocity is found during the workday in PG in the OE and CE conditions. One study analyzed the fatigue caused by work in firefighters and its relationship to balance, finding an increase in mean velocity in the measurement of post-fatigue, contrary to the findings in our study for PG [43]. In line with the previous study, there is also a report of an increase in COP velocity post-fatigue after walking and running [44] and following repeated plantar-flexion exercise [45]. A possible explanation for this difference is that the fatigue generated by intensive exercise or specific foot exercises is different from the activity done in assembly chains, which involves mainly simple and repetitive tasks done by the upper limbs and postural muscles. 

Several COP parameters could improve at the end of the workday after the deterioration suffered in the middle of the workday with the AP displacement in the OE condition, or on the contrary improve in the middle of the workday and deteriorate again at the end. Our theory for this fact is an adjustment of the body posture to the workload during the day. Work tasks in a manufacturing company can be considered as a prolonged non-intensive exercise that contributes to altering the effectiveness of sensory inputs and motor output of postural control. Compensatory postural strategies could be triggered to counteract the postural control due to the fatigue modifying the directions of the sway and modifying the strategies of balance control. 

### 4.3. Balance and Visual Feferences

Balance relies on three sensory systems: vestibular, proprioceptive, and visual. By challenging these systems, their contribution to balance control can be studied. In our study, only the visual condition is modified, capturing the balance with open and closed eyes. Analyzing the balance before the workday, a significant increase in AP, ML displacement, the sway area, and mean velocity was found comparing the open and closed-eyes conditions. 

The RMS-AP and RMS-ML also showed a significant increase in CE. Similar results were obtained by Brumagne et al. [46], where significantly more AP displacement was seen in the persons with recurrent LBP when vision was occluded. Vision could be a compensatory tool for balance control under pathologies that could alter proprioceptive inputs, such as LBP [14,15]. Yahia et al. [47] found an increase in the sway area and sway length in LBP patients between the open and closed-eyes conditions. NPG shows a decrease in the Romberg coefficient during the workday, which could be seen as a greater use of visual references for maintaining stability when fatigue is present. In PG, this decrease in the Romberg coefficient is not found. A possible explanation is that PG patients present altered proprioceptive information, relying for their balance primarily on the visual system. Although their static stability deteriorates during the workday, visual references are used with a similar contribution to maintain balance.

## 5. Conclusions

Analyzing the static balance of workers in a manufacturing plant with and without LBP, no significant differences were found in the balance parameters between the two groups of workers, although three variables (ML_OE, RMS_AP_OE, and RMS-ML_CE) present a medium effect size. According to the evolution of the COP during the workday, the ML displacement of COP in the open-eyes condition is the only variable that significantly decreases during the first part of the workday in PG workers. Due to the small sample size of the study, the expected changes in most of the COP parameters modified by the workday activity did not show statistically significant changes over the three measurements. However, the PG did not show a higher deterioration in static balance than NPG workers as hypothesized.

One limitation of our study is that the number of the workers in the NPG and PG were low and not homogeneous. A larger sample could help to clarify our hypothesis. Another limitation is that all the workers had previously taken sickness leave due to lumbar pain, but the researchers did not know the different diagnoses, which could have been useful for analyzing the data according to the previous pathology. Finally, we have considered only the previous workday level of LBP, but we had no data on the pain level at the end of the workday. This data would have given us information about whether the deterioration of static balance may be associated with an increase in the degree of LBP.

## Figures and Tables

**Table 1:** Basal characteristics of workers. Values are mean± SD. BMI: body mass index; LBP: low back pain. The symbol * denotes a significant difference between pain group (PG) and non-pain group (NPG) (*p* < 0.05)**.**

	Total (*n* = 22)	NPG (*n* = 5)	PG (*n* = 17)	*p*-Value
Age (years)	43 ± 7	48 ± 10	43 ± 5	0.145
Height (cm)	175.2 ± 8.9	169.2 ± 7.3	176.6 ± 8.7	0.138
Weight (kg)	80.8 ± 12.2	76.2 ± 14.5	81.9 ± 11.8	0.417
BMI (kg/m^2^)	26.3 ± 4.0	27.03 ± 7.3	26.23 ± 2.9	0.844
Current LBP	2.8 ± 1.8	0 ± 0	3.7 ± 0.9	0.002 *

**Table 2:** Center of pressure (COP) parameters before the workday in the non-pain group (NPG) and pain group (PG): antero-posterior with open eyes (AP_OE) and closed eyes (AP_CE); medial-lateral displacements with open eyes (ML_OE) and closed eyes (ML_CE); sway area with open eyes (Area_OE) and closed eyes (Area_CE); mean velocity with open eyes (MV_OE) and closed eyes (MV_CE); root mean square (RMS) in antero-posterior direction with open eyes (RMS_AP_OE) and closed eyes (RMS_AP_CE); RMS in medial-lateral direction with open eyes (RMS_ML_OE) and closed eyes (RMS_ML_CE); Romberg coefficient. Values are mean ± SD.

Group	NPG (*n* = 5)	PG (*n* = 17)	*p*-Value	ES
AP_OE(mm)	6.03 ± 1.75	6.77 ± 2.80	0.587	0.31
ML_OE (mm)	5.53 ± 1.58	9.20 ± 5.38	0.158	0.92
Area_OE (mm^2^)	26.40 ± 12.67	36.32 ± 26.29	0.431	0.48
MV_OE (mm/s)	5.22 ± 1.92	6.01 ± 2.02	0.449	0.40
RMS-AP_OE (mm)	0.38 ± 0.08	0.47 ± 0.15	0.200	0.74
RMS-ML_OE (mm)	0.35 ± 0.09	0.39 ± 0.12	0.520	0.37
AP_CE(mm)	9.76 ± 5.93	8.79 ± 4.00	0.674	0.19
ML_CE (mm)	8.88 ± 3.38	9.34 ± 3.55	0.799	0.13
Area_CE (mm^2^)	58.80 ± 46.62	51.93 ± 39.89	0.750	0.15
MV_CE (mm/s)	5.63 ±1.58	6.46 ± 1.75	0.357	0.49
RMS-AP_CE (mm)	0.53 ± 0.13	0.52 ± 0.11	0.963	0.08
RMS-ML_CE (mm)	0.39 ± 0.07	0.48 ± 0.19	0.300	0.62
Romberg	0.89 ± 0.12	0.87 ± 0.14	0.719	0.49
